# A Propensity-Matched Analysis of Survival of Clinically Diagnosed Early-Stage Lung Cancer and Biopsy-Proven Early-Stage Non-Small Cell Lung Cancer Following Stereotactic Ablative Radiotherapy

**DOI:** 10.3389/fonc.2021.720847

**Published:** 2021-08-24

**Authors:** Ran Zhang, Yanling Guo, Yujie Yan, Yuanjun Liu, Yaoyao Zhu, Jingjing Kang, Fangjuan Li, Xiaojiang Sun, Ligang Xing, Yaping Xu

**Affiliations:** ^1^Department of Radiation Oncology, Shanghai Pulmonary Hospital, School of Medicine, Tongji University, Shanghai, China; ^2^Tongji University, Shanghai, China; ^3^First Clinical Medical School, Wenzhou Medical University, Wenzhou, China; ^4^Department of Radiation Oncology, Cancer Hospital of University of Chinese Academy of Sciences (Zhejiang Cancer Hospital), Institute of Cancer and Basic Medicine (IBMC), Chinese Academy of Sciences, Hangzhou, China; ^5^Department of Radiation Oncology, Shandong Key Laboratory of Radiation Oncology, Shandong Cancer Hospital and Institute, Shandong First Medical University and Shandong Academy of Medical Sciences, Jinan, China

**Keywords:** stereotactic body radiotherapy, propensity-matched analysis, clinical diagnosis, early stage, lung cancer

## Abstract

**Purpose:**

Stereotactic body radiotherapy (SBRT) has been increasingly regarded as a reasonable option for early-stage lung cancer patients without pretreatment pathologic results, but the efficacy and safety in a Chinese population remains unclear. The aim of this study was to compare survival outcomes and toxicities between patients with clinically diagnosed early-stage lung cancer or biopsy-proven early-stage non-small cell lung cancer and to demonstrate the rationality of this treatment.

**Material and Methods:**

From May 2012 to December 2018, 56 patients with clinically diagnosed early-stage lung cancer and 60 patients with early-stage biopsy-proven were selected into non-pathological group and pathological group, respectively. Propensity score matching (PSM) was performed to reduce patient selection bias. Survival analysis with log-rank test was used to assess the differences of treatment outcomes, which included local control (LC), progression-free survival (PFS), and overall survival (OS).

**Results:**

The median age was 76 (range 47–93) years, and the median follow-up time was 58.3 (range 4.3–95.1) months in the cohort without pathologic results. The median age was 74 (range 57–88) years, and the median follow-up time was 56.3 (range 2.6–94) months in the cohort with pathologic results. 45 matched-pair were analyzed. The 5-year LC, PFS, and OS rates in matched-pair patients with or without pathologic biopsy were 85.5% and 89.8%, 40.6% and 70.9%, and 63.2% and 76.1%, respectively. On Kaplan-Meier survival analysis after PSM analysis, there was no significant difference between patients with pathologic results versus patients with no pathologic results in terms of LC (P= 0.498) and OS (P=0.141). Of the matched-pair patients treated with SBRT, only 1 patient experienced grade 3 or above radiation pneumonitis.

**Conclusion:**

For early-stage lung cancer patients with medically inoperable or not suitable for invasive diagnosis, SBRT may be a good local treatment.

## Introduction

Primary lung cancer, one of the most common neoplasms, is the leading cause of cancer-related deaths worldwide (1). In recent years, the widespread use of low-dose computerized tomography (LDCT) in clinical practice has led to a marked increase in the number of incidental findings of nodules suspicious for early-stage non-small cell lung cancer (NSCLC) (2), and this trend is expected to continue in the future. At present, surgical resection remains to the gold standard in the treatment of suitable patients with early stage NSCLC (3). As for those patients unsuited for surgery, stereotactic body radiotherapy (SBRT), a high-dose and precisely conformal radiation approach, can achieve superb local control and a sharp dose fall-off to surrounding crucial normal structures (4, 5), and has been proven to have comparable treatment results to lung resection in both its therapeutic and adverse effects (6).

Indeed, obtaining a pathological conformation prior to SBRT in patients with inoperable early stage NSCLC remains necessary in case of the inclusion of patients with benign lesions (7). However, a significant proportion of patients receiving SBRT are not suited for pretreatment biopsy diagnosis. For example, for patients with severe obstructive or restrictive lung disease, the risk of life-threatening hemoptysis or pneumothorax can impede a biopsy attempt. Moreover, pathological results before radiation are considered difficult to obtain especially in those elderly patients who are unwilling to have any invasive operation. For these patients without pathological biopsy, SBRT may possibly be regarded as an excessive medical treatment due to the possibility of benign nodules, but patients with untreated early-stage NSCLC have a relatively poor survival with a median survival time of 10 months, and a 5-year survival rate of only 2% (8). SBRT, a precise, noninvasive therapy, therefore, seems to be a reasonable solution for the empirical treatment of patients who cannot acquire pretreatment assurance of pathology.

SBRT without pretreatment tissue conformation for patients with early-stage NSCLC has also been recommended by recent national comprehensive cancer network (NCCN) guidelines (version 6.2020) which emphasized the importance of multidisciplinary discussion (9). However, there is still controversy over the survival benefit of SBRT for early-stage NSCLC patients with no pathological results as compared to pathologically confirmed patients. Recently, a meta-analysis reported by Michel et al. (10) found that survival outcomes are affected by a lack of biopsy confirmation and emphasized the importance of obtaining pathological proof before radiotherapy; however, this study included some patients for whom the diagnostic method was unclear, while the clinically diagnosed group had undergone surgical treatment, both of which may have influenced the statistical accuracy of the results. To date, few studies have been conducted to explore this issue, especially in the Chinese region where lung cancer is considered to be the leading cause of cancer-related death. Therefore, the aim of our study was to estimate and compare survival outcomes of SBRT in early-stage patients with or without pathological diagnosis by using the propensity score matching (PSM) to minimize the selection bias.

## Materials and Methods

### Patient Selection

**Figure 1:**
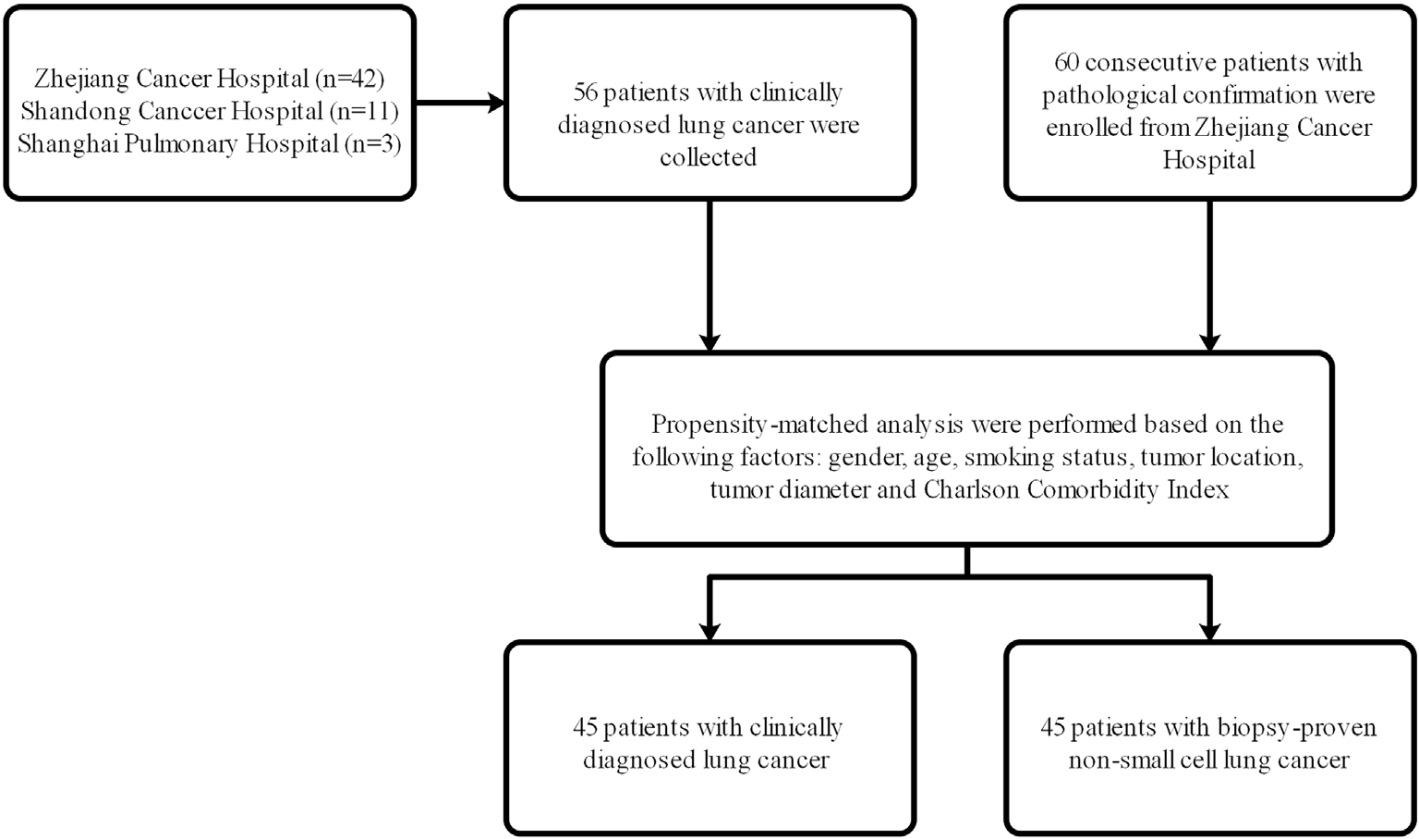
The flow chart of patient selection.

From May 2012 to December 2018, 56 patients with clinically diagnosed lung cancer who received SBRT in Shanghai Pulmonary Hospital, Shandong Cancer Hospital, and the Cancer Hospital of the University of Chinese Academy of Sciences (Zhejiang Cancer Hospital) were selected, and 60 consecutive patients with pathological confirmation who received SBRT in the Cancer Hospital of the University of Chinese Academy of Sciences (Zhejiang Cancer Hospital) were enrolled. The flow chart of patient selection was shown in **Figure 1**. Ethical approval was obtained at the institutional review board of the above-mentioned hospitals, and informed consent was waived from all patients due to the retrospective study. The indications for SBRT were clinically or pathologically confirmed early-stage NSCLC patients (stage T1-2N0M0) who refused surgery or who were inoperable. The eighth edition of the American Joint Committee on Cancer (AJCC)/tumor, node, metastasis (TNM) staging system was used in all patients of this study. Based on the clinical, laboratory, and radiology data, the clinical diagnosis of lung cancer and treatment of SBRT for the group without pathological biopsy was confirmed by a multidisciplinary tumor board consisting of radiologists, thoracic surgeons and radiation oncologists. The inclusion criteria of patients without biopsy confirmation treated with SBRT were as follows: (1) positive imaging features of malignancies, such as progressive enlargement of lesions, the increase in the density or proportion of ground-glass opacity (GGO), or the appearance of vascular perforation and spiculation signs at the edge in contrast-enhanced CT or 1-3 mm thin-section CT; (2) positive lesions in positron emission tomography/computed tomography (PET/CT); (3) consensus of the multidisciplinary team of lung cancer; and (4) agreement of patients and their family members. The concrete information on PET/CT or CT of these patients was presented in **Supplementary Table 1**. Those patients who had chemotherapy or radiotherapy before SBRT, who presented with metastatic tumors and who had diagnosis of interstitial lung disease (ILD) were excluded from this study.

### SBRT Treatment

SBRT delivery and planning were performed as described in our previous studies (11, 12). Briefly speaking, the patients were immobilized in a vacuum bag and underwent stimulation with 4-dimensional computed tomography (4D-CT). If the tumor movement was greater than 1.5 cm, abdominal compression was used. Before each fraction, respiratory gating and cone beam CT were implemented for image-guided treatment. The gross tumor volume (GTV) was delineated on the basis of CT scanning by maximal intensity projection in the lung window, and the internal tumor volume (ITV) was generated according to the target motion by 4D-CT in most patients. Finally, a 5-mm setup margin was added to the ITV for creating the planned target volume (PTV), and no clinical target volume (CTV) was generated. Therapeutic plans were optimized to limit high doses and protect organs at risk, such as the healthy lung, trachea, proximal bronchial tree, spinal cord, esophagus, great vessels, heart, and chest wall. Treatment was delivered using a 6-MV or 10-MV linear accelerator. The 80% isodoses were chosen as dose prescription to ensure 95%, 99% and 100% of the PTV, ITV and GTV, respectively. The median number of fractions was 5 (3–12). The median dose of SBRT was 50 Gy (40–70 Gy). The median biologically effective dose (BED) was 100Gy (71.4–119 Gy). In order to calculate the BED, we defined the value of α/β as 10 (13).

### Follow-Up and Outcome Assessment

After the treatment of SBRT, the first follow-up was evaluated by routine blood test, B-ultrasonography, and CT scanning. We then performed these examinations every 3 months for 2 years, and every 6 months thereafter. PET/CT was used when tumor relapse or metastasis were highly suspected. Those patients who received routine follow-up at local hospitals were evaluated by treating doctors and contacted by phone. The infield effect was assessed in light of the Response Evaluation Criteria in Solid Tumors (RECIST), version 1.1, and therapeutic toxicity was evaluated according to the Common Terminology Criteria for Adverse Events (CTCAE), version 5.0. Local control (LC), progression-free survival (PFS), and overall survival (OS) were correlated with local failure, disease progression or death, and death, respectively. The duration of follow-up was defined as the time between the first day of radiation and the data of endpoints or last follow-up.

### Statistical Analysis

In order to reduce the patients’ selection bias, a PSM analysis was utilized to match baseline characteristics between the pathologically confirmed group and non-pathologically confirmed group. Patients were matched in light of these factors: gender, age, smoking status, tumor location, tumor diameter and Charlson Comorbidity Index (CCI). Baseline characteristics in both groups were compared using the Mann-Whitney U test or chi-square test. The differences of treatment outcomes, including LC, PFS and OS, were analyzed by Kaplan-Meier survival curves with log-rank tests, and a life table was used to calculate the concrete 1-year, 3-year, or 5-year rates of LC, PFS and OS. For all tests, a P value of <0.05 was deemed significant. Statistical analyses were performed with SPSS software (version 22.0, IBM Corp.) and GraphPad Prism (version 8.0.2, GraphPad Software Inc.).

## Results

### Patient Characteristics

For this analysis, 56 patients with clinically diagnosed stage I lung cancer and 60 patients with pathological confirmation were recruited into this study. The details of patients’ characteristics for both groups are shown in **Table 1**. For patients with clinical lung cancer, the median age was 76 (range 47–93) years, and the median follow-up time was 58.3 (range 4.3–95.1) months. For patients with pathological NSCLC, the median age was 74 (range 57–88) years, and the median follow-up time was 56.3 (range 2.6-94) months. With regard to age, gender, median follow-up time, smoking status, inoperable status, tumor diameter, tumor location, T stage, histology, global initiative for chronic obstructive lung disease (GOLD) score, eastern cooperative oncology group (ECOG) performance status score, charlson comorbidity index (CCI) and biological effective dose (BED), both groups were well balanced.

**Table 1:** Characteristics of all patients.

	Pathological NSCLC, n (%)	Clinical NSCLC, n (%)	*P* value
**Number of patients**	60	56	N.A.
**Median follow-up (months, range)**	56.3 (2.6-94)	58.3 (4.3-95.1)	0.571
**Median age (range)**	74 (57-88)	76 (47-93)	0.507
**Gender**			0.794
Male	45 (75.0%)	34 (60.7%)	
Female	15 (25.0%)	22 (39.3%)	
**Smoking**			0.825
Yes	41 (68.3%)	31 (55.4%)	
No	19 (31.7%)	25 (44.6%)	
**GOLD**			0.389
0	29 (48.3%)	25 (44.6%)	
I	4 (6.6%)	5 (8.9%)	
II	19 (31.7%)	20 (35.7%)	
III	8 (13.4%)	6 (10.8%)	
IV	0 (0.0%)	0 (0.0%)	
**ECOG performance score**			0.797
0	12 (20.0%)	13 (23.2%)	
1	45 (75.0%)	35 (62.5%)	
2	3 (5.0%)	7 (12.5%)	
3	0 (0%)	1 (1.8%)	
**CCI**			0.999
0-5	59 (98.3%)	53 (94.6%)	
6	1 (0.7%)	3 (5.4%)	
**Inoperable**			0.645
Yes	49 (81.6%)	40 (71.4%)	
No	11 (18.4%)	16 (28.6%)	
**Diameter (median, range)**	2.2 (0.7-4.7)	1.9 (0.5-4.0)	0.009
**Location: central/peripheral**	3 (5.5%)/57 (94.5%)	2 (3.6%)/54 (96.4%)	0.275
**T stage**			0.115
T1a	1 (1.7%)	7 (12.5%)	
T1b	28 (46.7%)	29 (51.8%)	
T1c	21 (35.0%)	17 (30.4%)	
T2a	7 (11.7%)	3 (5.3%)	
T2b	3 (4.9%)	0 (0%)	
**Histology**			N.A.
Adenocarcinoma	29 (48.3%)	0 (0.0%)	
Squamous cell ca	17 (28.3%)	0 (0.0%)	
Unclassified NSCLC	14 (23.4%)	0 (0.0%)	
Histologically unproven	0 (0.0%)	55 (100%)	
**BED**			0.999
≥100	41 (68.3%)	41 (73.2%)	
<100	19 (31.7%)	15 (26.8%)	

NSCLC, non-small cell lung cancer; GOLD, Global Initiative for Chronic Obstructive Lung Diseases stage; ECOG, eastern cooperative oncology group; CCI, Charlson Comorbidity Index; BED, biologically effective dose; N.A., not applicable.

After PSM analysis, 45 patients were identified from each group. The details of patients’ characteristics for both groups after PSM analysis are shown in **Table 2**. For patients with clinical lung cancer, the median age was 76 (range 47–88) years, and the median follow-up time was 60.6 (range 8.2–95.1) months. For patients with pathological NSCLC, the median age was 75 (range 57–88) years, and the median follow-up time was 55.2 (range 2.7-94) months. With regard to age, gender, median follow-up time, smoking status, inoperable status, tumor diameter, tumor location, T stage, histology, global initiative for chronic obstructive lung disease (GOLD) score, eastern cooperative oncology group (ECOG) performance status score, Charlson Comorbidity Index (CCI) and biological effective dose (BED), both groups were well balanced.

**Table 2:** Characteristics of propensity score matched patients.

	Pathological NSCLC, n (%)	Clinical lung cancer, n (%)	*P* value
**Number of patients**	45	45	N.A.
**Median follow-up (months, range)**	55.2 (2.7-94)	60.6 (8.2-95.1)	0.862
**Median age (range)**	75 (57-88)	76 (47-88)	0.859
**Gender**			0.569
** Male**	31 (68.9%)	30 (66.7%)	
** Female**	14 (31.1%)	15 (33.3%)	
**Smoking**			0.406
** Yes**	28 (62.2%)	26 (57.8%)	
** No**	17 (37.8%)	19 (42.2%)	
**GOLD**			0.188
** 0**	20 (44.4%)	18 (44.6%)	
** I**	4 (8.9%)	4 (8.9%)	
** II**	14 (31.1%)	18 (35.7%)	
** III**	7 (15.6%)	5 (10.8%)	
** IV**	0 (0.0%)	0 (0.0%)	
**ECOG performance score**			0.952
** 0**	9 (20.0%)	11 (24.4%)	
** 1**	34 (75.6%)	29 (64.4%)	
** 2**	2 (4.4%)	4 (8.9%)	
** 3**	0 (0%)	1 (2.3%)	
**CCI**			0.99
** 0-5**	44 (97.8%)	44 (97.8%)	
** 6**	1 (2.2%)	1 (2.2%)	
**Inoperable**			0.645
** Yes**	37 (82.2%)	32 (71.1%)	
** No**	8 (17.8%)	13 (28.9%)	
**Diameter (median, range)**	2.0 (0.7-3.8)	1.7 (0.5-4.0)	0.177
**Location: central/peripheral**	2 (4.4%)/43 (95.6%)	2 (4.4%)/43 (95.6%)	0.999
**T stage**			0.453
** T1a**	1 (2.2%)	4 (8.9%)	
** T1b**	22 (48.9%)	25 (55.6%)	
** T1c**	18 (40.0%)	13 (28.9%)	
** T2a**	4 (8.9%)	3 (6.6%)	
** T2b**	0 (0%)	0 (0%)	
**Histology**			N.A.
**Adenocarcinoma**	23 (51.1%)	0 (0.0%)	
**Squamous cell ca**	9 (20.0%)	0 (0.0%)	
**Unclassified NSCLC**	13 (28.9%)	0 (0.0%)	
**Histologically unproven**	0 (0.0%)	55 (100%)	
**BED**			0.140
** ≥100**	30 (66.7%)	34 (75.6%)	
** <100**	15 (33.3%)	11 (24.4%)	

NSCLC, non-small cell lung cancer; GOLD, Global Initiative for Chronic Obstructive Lung Diseases stage; ECOG, eastern cooperative oncology group; CCI, Charlson Comorbidity Index; BED, biologically effective dose; N.A., not applicable.

### Survival Outcomes

**Figure 2:**
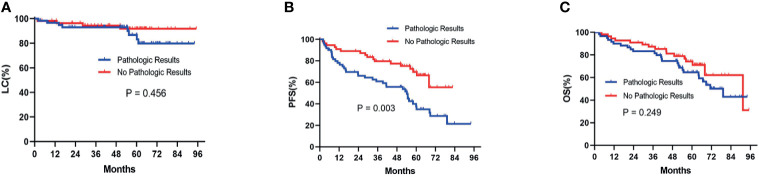
Kaplan-Meier survival analysis before the PSM analysis for LC **(A)**, PFS **(B)**, and OS **(C)** between patients with pathologic results and patients with no pathologic results. PSM, propensity score matching; LC, local control; PFS, progression-free survival; OS, overall survival.

**Figure 3:**
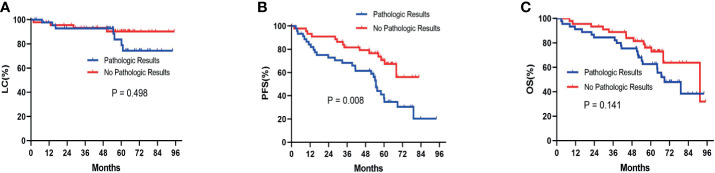
Kaplan-Meier survival analysis after the PSM analysis for LC **(A)**, PFS **(B)**, and OS **(C)** between patients with pathologic results and patients with no pathologic results. PSM, propensity score matching; LC, local control; PFS, progression-free survival; OS, overall survival.

Before the PSM analysis, Kaplan-Meier survival analysis showed that there was no significant difference between patients with pathologic results versus patients with no pathologic results in terms of LC (P= 0.456) (**Figure 2A**) and OS (P= 0.249) (**Figure 2C**). With respect to PFS, patients without pathologic results were proven to have a significantly better performance than patients with pathologic results (P= 0.003) (**Figure 2B**). After performing PSM analysis, similarly, Kaplan-Meier survival analysis demonstrated that there was no significant difference between patients with pathologic confirmation versus patients with no pathologic confirmation in terms of LC (P= 0.498) (**Figure 3A**) and OS (P= 0.141) (**Figure 3C**). With regard to PFS, patients without pathologic results were proven to have a significantly better performance than patients with pathologic results (P= 0.008) (**Figure 3B**). The pretreatment and posttreatment performance score (PS) of all patients from both cohorts were estimated and demonstrated to have no significant variance before the PSM analysis (P= 0.119) or after the PSM analysis (P= 0.399).

The outcomes comparison of the matched-pair groups is summarized in **Table 3**. The median OS of patients with or without biopsy confirmation was 68.7 and 91.4 months, respectively. The median PFS of patients with biopsy results was 54.7 months, and the median PFS of patients without biopsy results was not available. For the 45 patients with biopsy results, there were 12 patients who were died of tumor, 4 patients who were died of multiple organ dysfunction, 3 patients died of cerebral infarction, 1 patient who was died of heart failure, and 1 patient who was died of asthma. For the 45 patients with clinically diagnosed lung cancer, there were 6 patients who were died of tumor, 5 patients who were died of multiple organ dysfunction, 1 patient who were died of respiratory failure, and 1 patient who were died of cardiopulmonary dysfunction.

**Table 3:** Summary and comparison of outcomes with long-term follow-up in patients with pathological NSCLC or clinical lung cancer after PSM analysis.

	Pathological NSCLC	Clinical lungcancer
**Local control rate, % (95% CI)**		
1 year	97.7 (93.2-100)	97.8 (93.4-100)
3 year	92.6 (84.5-100)	93.0 (85.4-100)
5 year	85.5 (73.4-97.5)	89.8 (80.2-99.4)
**OS rate, % (95% CI)**		
Number of deaths	21	13
1 year	91.1 (82.8-99.4)	95.6 (89.5-100)
3 year	84.4 (73.9-95.0)	88.8 (79.5-98.1)
5 year	63.2 (48.7-77.6)	76.1 (63.0-89.1)
Median, months	68.7	91.4
**PFS rate, % (95% CI)**		
Number of failures	28	14
1 year	84.3 (73.6-95.0)	93.3 (85.9-100)
3 year	68.3 (54.6-82.1)	81.7 (70.2-93.2)
5 year	40.6 (25.4-55.7)	70.9 (56.8-84.9)
Median, months	54.7	N.A.

N.A., not applicable.

By using the PSM analysis, the 3-year LC, PFS, and OS rates in patients with or without pathologic biopsy were 92.6% and 93.0%, 68.3% and 81.7%, and 84.4% and 88.8%, respectively. The 5-year LC, PFS, and OS rates in patients with or without pathologic biopsy were 85.5% and 89.8%, 40.6% and 70.9%, and 63.2% and 76.1%, respectively.

### Toxicity

The treatment of radiation was well tolerated in both cohorts identified by PSM analysis. The results of radiation pneumonitis grade are listed in **Table 4**. Among the biopsy-proven group, grade 2 radiation pneumonitis was identified in 5 patients (11.1%). Among the nonpathologically proven group, grade 2 radiation pneumonitis was identified in 4 patients (9.0%). One patient in the biopsy-proven group experienced grade 5 radiation pneumonitis. No grade 4 or 5 radiation pneumonitis occurred in patients without pathological results, and no other toxicities (such as rib fractures, radiation esophagitis, and chest pain) of grade 3 or above were observed.

**Table 4:** Adverse effects related to SBRT following PSM analysis.

RP grade	Pathological NSCLC, n (%)	Clinical NSCLC, n (%)
0	24 (53.3%)	29 (64.4%)
1	15 (33.3%)	12 (26.6%)
2	5 (11,1%)	4 (9.0%)
3	0 (0%)	0 (0%)
4	0 (0%)	0 (0%)
5	1 (2.3%)	0 (0%)
**Rib fracture grade**		
0	45 (100%)	45 (100%)
**RE grade**		
0	43 (95.5%)	43 (95.5%)
1	2 (4.5%)	2 (4.5%)
**Chest pain grade**		
0	41 (91.1%)	44 (97.8%)
1	4 (8.9%)	1 (2.2%)

SBRT, stereotactic body radiotherapy; RP, radiation pneumonitis; NSCLC, non-small cell lung cancer, RE, radiation esophagitis.

### Performance Scores

**Figure 4:**
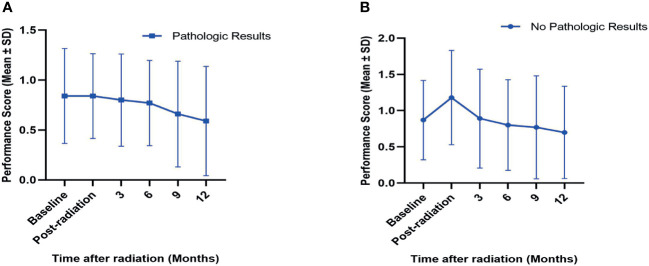
Variations (mean ± SD) in performance scores in SBRT-treated patients with **(A)** or without **(B)** pretreatment pathologic results after PSM analysis. The performance scores for baseline; post-radiation; and 3, 6, 9, and 12 months after SBRT in both cohorts are presented. SD, standard deviation; PSM, propensity score matching.

All patients in both cohorts received the evaluations of performance score by the treating doctors. For the matched-pair groups, performance scores before radiation, after radiation, at 3, 6, 9, and 12 months after radiation were recorded, along with those patients who died and for whom complete evaluations after death could not be completed. The mean performance scores in groups with or without pathologic results at baseline; posttreatment; and 3, 6, 9, and 12 months after radiation are presented in **Figure 4**. A trend of a gradual decrease in the performance scores in both the pathological cohort and nonpathological cohort was observed, and these data indicated that SBRT improved the quality of life in spite of its toxicity.

## Discussion

The main finding of this retrospective research was that there were no significant differences in LC and OS after SBRT for early-stage NSCLC patients with or without pretreatment pathologic results. Patients with no pathological confirmation achieved satisfactory outcomes and had comparable rates of LC and OS compared with the rates of patients with biopsy confirmation. The LC rates at 5 years’ follow-up in the nonpathological group exceeded 90%, and this was similar to the long-term results of the RTOG 0236 trial with a 92.7% LC rate (14). Although there were several other competing causes of deaths for medically inoperable patients (15), the OS rates in both groups had satisfactory outcomes and were 59.0% and 64.6%, respectively. Equally crucial is that few high-grade toxicities were shown in both cohorts of our study, which was in accordance with the results of previous studies (16–18). In addition, all performance scores estimated in this research were also satisfactory, and this also indirectly indicated good survival quality after SBRT treatment.

Given that the above-mentioned clinical results were positive, it is reasonable that SBRT should be regarded as an option for early-stage NSCLC without tissue biopsy. As promising rates of LC and OS have been reported in recent years (19, 20), a growing body of studies have begun to explore a new patient population suitable for the treatment of SBRT, including operable patients for whom surgery may be risky (21–23) and patients with central early-stage NSCLC in whom it is difficult to operate (11). The histologically unproven patients with early-stage lung cancer have also been considered candidates for SBRT. Previous studies (17, 18, 24) demonstrated similar outcomes of LC, OS, and even PFS among patients with no tissue confirmation relative to biopsy-confirmed patients. The use of SBRT for early-stage lung cancer in China has not been popularized, and thus there have been few studies reporting the outcomes concerning the therapeutic method of SBRT in a Chinese population for patients without tissue diagnosis. Our study provides further relevant data in this field, and additional studies in other cities and hospitals are needed to validate our results.

Indeed, administering SBRT for early-stage lung cancer patients without a pretreatment biopsy confirmation may increase the risk of inclusion of patients with no malignancies. Due to the inevitable possibilities of enrolling patients with benign nodules, the survival outcomes of the group with no tissue biopsy before radiation treatment might be overestimated. In our study, the Kaplan-Meier survival analysis of PFS showed that the group without pathologic results had significantly better performances than the group with pathologic results, and even the outcomes of OS in the biopsy-unproven cohort, although not significant, were slightly higher than those in patients with pathological biopsy. These results of course indicate an increased possibility of including nodules highly suspicious for benign lesions. The main problems of inclusion of patients with benign nodules is excessive therapy and unnecessary damage to healthy lung tissues. However, in our research, the tolerated toxicities and good performance scores in the tissue-unproven arm suggest that the treatment of SBRT had little influence on the quality of life even in patients with no malignancies. It has been reported that an increasing number patients without a preceding biopsy are undergoing SBRT as a therapeutic regimen (25). Therefore, considering SBRT as an effective treatment for patients for whom obtaining biopsy constitutes a health risk is reasonable.

The main issue with using SBRT to cure patients with no pathological results is the discrimination of malignant nodules before radiation therapy. Establishing a predictive model of benign lesions may be an efficacious solution and a direction of future research. Several calculated models reported in previous studies, including the model established by Swensen et al. (26), have proven effective in predicting benign nodules. Yet, this model cannot replace gold standard biopsy diagnosis (27). Thus, a novel approach with multidimensional features is needed to upgrade diagnostic effectiveness, especially in the era of personalized medicine. Radiomics, which can reveal phenotypic information of tumors, may possibly be a noninvasive and easy-to-use method tailored to construct a model for precise diagnosis (28). Recently, deep learning models have shown strongly diagnostic abilities in several studies (29, 30), and may be considered as a substitutional pretreatment approach of biopsy in the future.

Because of the fact that we did not perform pretreatment pathologic check for those patients with a clinically diagnosed lung cancer, it was indeed possible that we may include some patients with early-stage patients with small-cell lung cancer (SCLC). However, for the patients with lung cancer, only 10-15% of patients were diagnosed as SCLC (31, 32). Early-stage SCLC (T1-2N0SCLC) comprises 7% of all SCLC and only 0.29% of all lung cancers (33).Therefore, the probability for us to include early-stage patients with SCLC was very small. In addition, those patients with early-stage SCLC treated by SBRT can also achieved a well-tolerated treatment with acceptable OS and excellent LC. Recently, there were an increasing of evidences for radiation oncologists to choose SBRT as reasonable treatment for patients with early-stage SCLC (34). Raj et al. reported that for the patients with early-stage SCLC following SBRT, the 1-year and 2-year OS were 73.1% and 36.6%, respectively, and the 1-, 2-, and 3-year LC rates for the cohort were 100% (35). In the future, Radiomics model may provide an effective method to distinguish between NSCLC and SCLC clinically (36).

In this study, we reported good performance in clinical outcomes and only mild toxicity in early-stage lung cancer patients without pathology diagnosis, with comparable outcomes of LC and OS as in pathologically proven patients. This preliminarily demonstrates the validity and justification for treating Chinese biopsy-unproven patients with SBRT. The above-mentioned results support the view that SBRT is suitable for prolonging survival time in the majority of nonpathology-proven patients as compared to a watch-and-wait approach. Our study provides further evidence supporting a noninvasive option of treatment for early-stage lung cancer patients who cannot obtain pathological confirmation prior to SBRT.

There are some limitations in this study. First, due to the retrospective nature of this study, though we utilized the PSM analysis to minimize the patient selection, there was an inevitable bias in this research. Thus, prospective randomized trials are still need in the future. Second, we did not use a quantitative model to identified the patients with clinically diagnosed early-stage lung cancer, but all these patients were strictly included according to the inclusion criterion and consensus of the multidisciplinary team of lung cancer. Third, in the comparison with the nonpathologically confirmed group, no control group of patients who do not receive any treatment was set to evaluate the benefit of SBRT, and thus further studies with a control group are needed to validate the results of this study.

## Conclusions

Our study provides further evidence for treating early-stage Chinese lung cancer patients without preceding pathologic results with SBRT, as shown by the satisfactory survival outcomes and mild toxicities in patients without pathologic results. For early-stage lung cancer patients with medically inoperable or not suitable for invasive diagnosis, SBRT may be a good local treatment.

## Data Availability Statement

The data generated for this study are available on request to the corresponding authors.

## Ethics Statement

The studies involving human participants were reviewed and approved by The ethics committee of Shanghai Pulmonary Hospital, Shandong Cancer Hospital, and the Cancer Hospital of the University of Chinese Academy of Sciences (Zhejiang Cancer Hospital) approved the study. Written informed consent for participation was not required for this study in accordance with the national legislation and the institutional requirements.

## Author Contributions

RZ performed the study and drafted the manuscript. YX, LX XS, and FL designed the study and JK helped to draft the manuscript. RZ, YG, YY, YL, and YZ helped to acquire the clinical data and image. RZ and YG offered assist for analysis of data. YX evaluated data. All authors contributed to the article and approved the submitted version.

## Funding

This work was supported by the Start-up Fund for Talent Introduction of Shanghai Pulmonary Hospital (YX) (grant number: 20180101) and the Science Research Foundation of China Ministry of Health - Zhejiang Medicine & Health Key Research Fund (no. 201339868).

## Conflict of Interest

The authors declare that the research was conducted in the absence of any commercial or financial relationships that could be construed as a potential conflict of interest.

## Publisher’s Note

All claims expressed in this article are solely those of the authors and do not necessarily represent those of their affiliated organizations, or those of the publisher, the editors and the reviewers. Any product that may be evaluated in this article, or claim that may be made by its manufacturer, is not guaranteed or endorsed by the publisher.
