# Supplementary material for: A Propensity-Matched Analysis of Survival of Clinically Diagnosed Early-Stage Lung Cancer and Biopsy-Proven Early-Stage Non-Small Cell Lung Cancer Following Stereotactic Ablative Radiotherapy
Source: Front Oncol. 2021 Aug 24;11:720847. doi: 10.3389/fonc.2021.720847 (PMC8421845; doi:10.3389/fonc.2021.720847)
Supplement: Supplementary file 1 [file Table_1.docx]

**Supplementary Table 1** Information on the characteristics of patients with clinically diagnosed early-stage lung cancer.

| **Characteristics** | **Clinical lung cancer, n** |
| --- | --- |
| **PET/CT (positive lesions)** | 42 |
| **CT** |  |
| progressive enlargement of lesions | 3 |
| increase in the density or proportion of GGO | 6 |
| appearance of vascular perforation | 2 |
| appearance of spiculation signs | 2 |

Abbreviations: PET/CT, positron emission tomography/computed tomography; CT, computed tomography.

**Supplementary Table 2** Tumor marker information of all patients.

|  | **Pathological NSCLC, n (%)** | **Clinical lung cancer, n (%)** | ***P* value** |
| --- | --- | --- | --- |
| **CEA positive** |  |  | 0.999 |
| Yes | 10 | 4 |  |
| No | 50 | 52 |  |
| **CYfra21-1 positive** |  |  | 0.772 |
| Yes | 29 | 20 |  |
| No | 31 | 36 |  |
| **NSE positive** |  |  | 0.999 |
| Yes | 4 | 1 |  |
| No | 56 | 55 |  |

Abbreviations: NSCLC, non-small cell lung cancer; CEA, carcinoembryonic antigen; NSE, neuron-specific enolase.
